# Supplementary material for: Mice Lacking the Systemic Vitamin A Receptor RBPR2 Show Decreased Ocular Retinoids and Loss of Visual Function
Source: Nutrients. 2022 Jun 8;14(12):2371. doi: 10.3390/nu14122371 (PMC9231411; doi:10.3390/nu14122371)
Supplement: Supplementary file 1 [file nutrients-14-02371-s001.zip › nutrients-1717118-supplementary.pdf]

**Supplementary Information**

# Mice lacking the systemic vitamin A receptor RBPR2 show decreased ocular retinoids and loss of visual function

Rakesh Radhakrishnan[1], Matthias Leung[1], Heidi Roehrich[1], Stephen Walterhouse[2], Altaf A. Kondkar[3], Wayne Fitzgibbon[2], Manas R. Biswal[4] and Glenn P. Lobo[1,2,5*]

[1] Department of Ophthalmology and Visual Neurosciences, University of Minnesota, Lions Research Building, 2001 6th Street SE, Minneapolis, MN 55455, USA.
[2] Department of Medicine, Medical University of South Carolina, Charleston, SC 29425, USA.
[3] Department of Ophthalmology and Glaucoma Research Chair, College of Medicine, King Saud University, Riyadh, Saudi Arabia.
[4] Department of Pharmaceutical Sciences, Taneja College of Pharmacy, University of South Florida, Tampa, FL 33612, USA.
[5] Department of Ophthalmology, Medical University of South Carolina, Charleston, SC 29425, USA.

*Correspondence: lobo0023@umn.edu

***Mouse*-RBPR2-Peptide1**

Sequence alignment shows the peptide1 alignment with mouse RBPR2 residue site 213, known for Cytoplasmic region according to Uniprot database

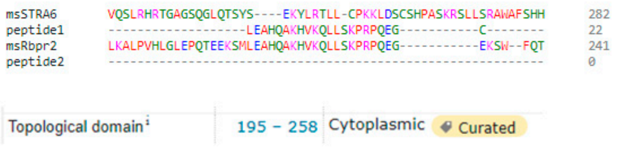


***Mouse*-RBPR2-Peptide2**

Sequence alignment shows the peptide2 alignment with mouse RBPR2 residue site 529, known for Extracellular region according to Uniprot database

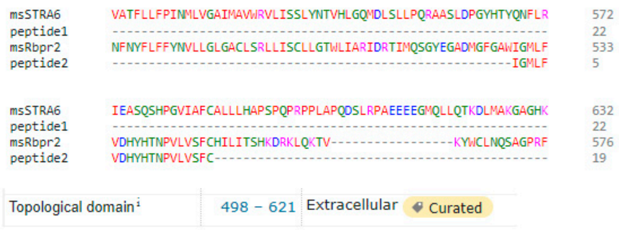


**Supplementary Figure S1: Mouse RBPR2 peptide 1 and 2 information.** Shown is the protein sequences of RBPR2 peptides 1 (cytoplasmic region) and 2 (extracellular region) used to generate the RBPR2 antibody and alignment between mouse RBPR2 and mouse STRA6 protein sequences, at the respective RBPR2 peptide positions.

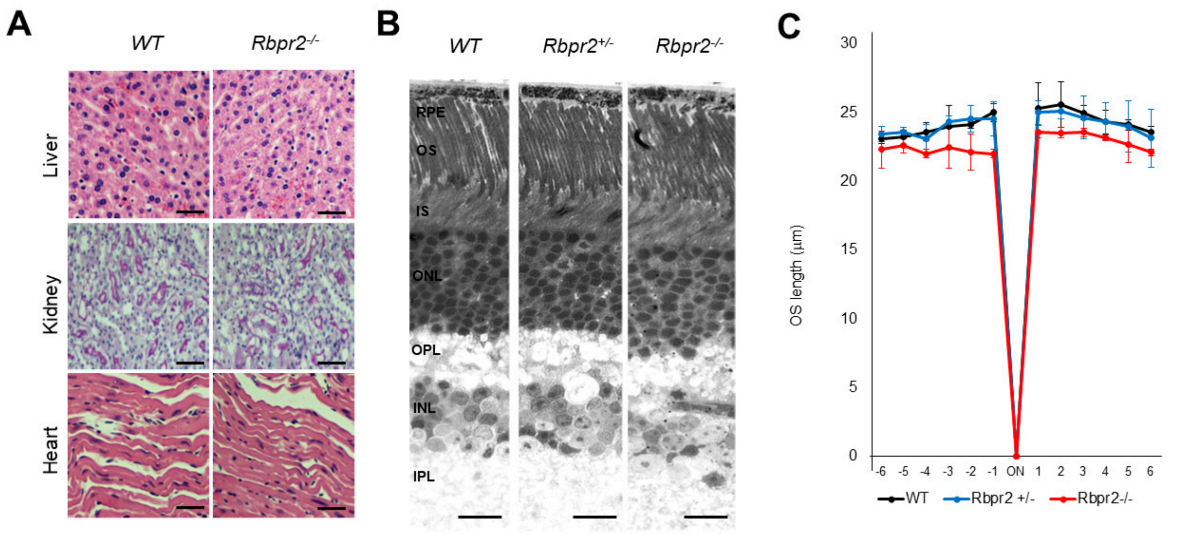


**Supplementary Figure S2: Histology of tissues from *Rbpr2*-/- and control mice maintained on vitamin A-sufficient diet. (A)** Representative tissue histology and staining of the liver, kidney (Periodic acid Schiff stain), heart, and **(B)** retinas (H&E stain) of 12-week old wild-type (WT) and homozygous *Rbpr2*$^{-/-}$ mice. **(C)** Quantification of photoreceptor outer segment (OS) lengths from H&E sections of 12-week old WT, *Rbpr2*$^{+/-}$, and *Rbpr2*$^{-/-}$ mice, using "spider graph" morphometry. The OS lengths from H&E sections through the optic nerve (ON; 0 µm distance from the optic nerve and starting point) were measured at 12 locations around the retina, six each in the superior and inferior hemispheres, each equally at approximately 150 µm distances. RPE, retinal pigmented epithelium; OS, outer segments; IS, inner segments; ONL, outer nuclear layer; INL, inner nuclear layer; IPL, inner plexiform layer; RE, retinyl esters; a*t*-ROL, all-*trans* retinol. Scale bar = 50 µm (**A**); 100 µm (**B**).

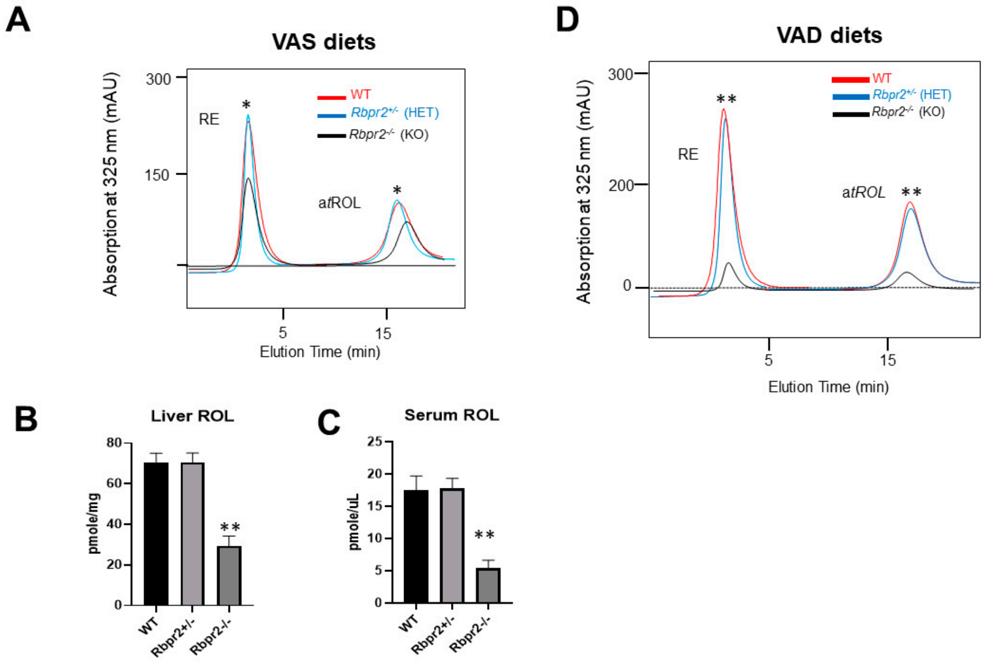


**Supplementary Figure S3: Representative HPLC traces of ocular retinoid content of eyes, non-ocular tissue, and serum.** HPLC traces from WT (red), heterozygous *Rbpr2*$^{+/-}$ (blue), and homozygous *Rbpr2*$^{-/-}$ (black) mice, from animals on vitamin A sufficient (**A**) or vitamin A deficient (**D**) diets. *$P$<0.05; **$P$<0.005; *Rbpr2*$^{-/-}$ compared to controls. (**B, C**) Liver and Serum ROL levels in *Rbpr2*$^{-/-}$ and control mice on VAS diets. RE, retinyl esters; a*t*ROL, all-*trans* retinol.

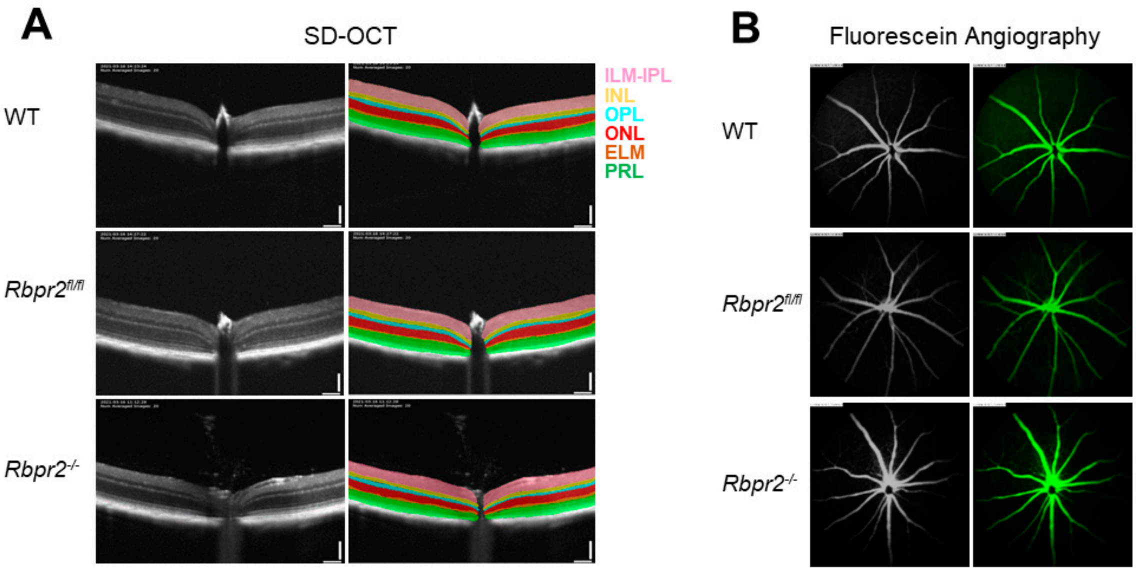


**Supplementary Figure S4: Non-invasive retinal analysis of *Rbpr2*$^{-/-}$ mice on vitamin A-sufficient diets. (A)** OCT retinal analysis of 12-week old control (WT, *Rbpr2*$^{fl/fl}$) and *Rbpr2*$^{-/-}$ mice on vitamin A sufficient diets. **(B)** Fundus imaging of 12-week old wild-type (WT), floxed (*Rbpr2*$^{fl/fl}$), and homozygous (*Rbpr2*$^{-/-}$) mice after intraperitoneal injection with ICG (15 mg/kg, Acros Organics, NJ, USA). PRL, photoreceptor layer; ELM, external limiting membrane; ONL, outer nuclear layer; OPL, outer plexiform layer; INL, inner nuclear layer; ILM-IPL, inner limiting membrane-Inner plexiform layer.

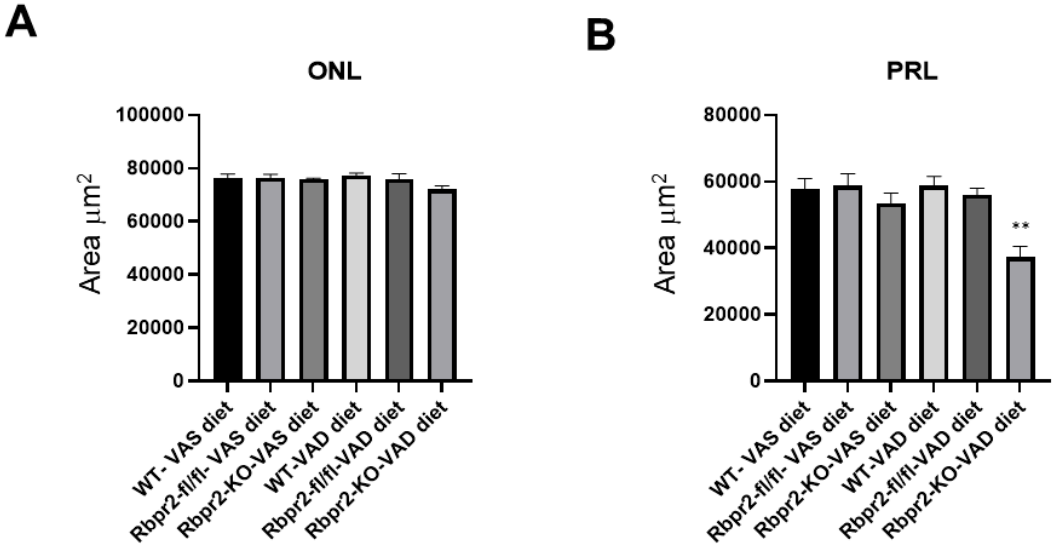


**Supplementary Figure S5: Quantification of photoreceptor cell length and outer nuclear layer thickness in retinas of control and *Rbpr2*$^{-/-}$ mice.** Control and *Rbpr2*$^{-/-}$ mice on either vitamin A sufficient (VAS) or vitamin A depleted (VAD) diets were subjected to OCT analysis (from **Figure 6A and Supplementary Figure S3A**) respectively. Image *J* software was used for quantification of outer nuclear layer (ONL) thickness (**A**) and photoreceptor cell layer (PRL) thickness (**B**). $^{**}P<0.005$.

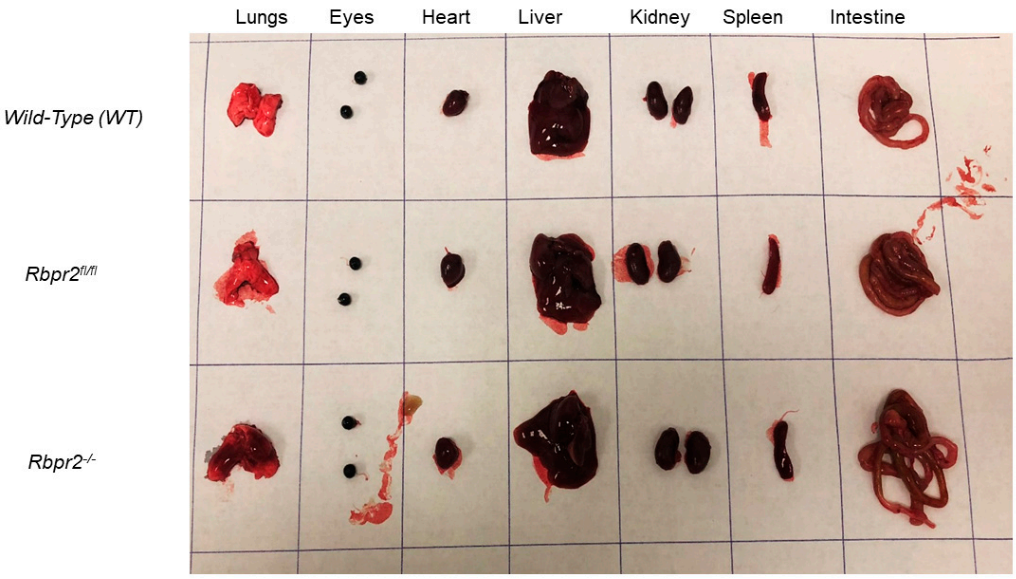


**Supplementary Figure S6: Gross pathology of tissues harvested from controls and *Rbpr2*$^{-/-}$ mice on vitamin A deficient diets.** Respective tissues were harvested from 12-week old control and *Rbpr2*$^{-/-}$ mice maintained on VAD diets and photographed, before histology. Data is representative of *n*=6 animals for each genotype.
